# Unraveling the Pathways to Neuronal Homeostasis and Disease: Mechanistic Insights into the Role of RNA-Binding Proteins and Associated Factors

**DOI:** 10.3390/ijms19082280

**Published:** 2018-08-03

**Authors:** Stylianos Ravanidis, Fedon-Giasin Kattan, Epaminondas Doxakis

**Affiliations:** Basic Sciences Division I, Biomedical Research Foundation, Academy of Athens, 11527 Athens, Greece; sravanidis@bioacademy.gr (S.R.); fkattan@bioacademy.gr (F.-G.K.)

**Keywords:** RNA-binding proteins, neurodegeneration, stress granules, RNP granules, Pumilio, Staufen, IGF2BP, FMRP, Sam68, CPEB, NOVA, ELAVL, SMN, TDP43, FUS, TAF15, TIA1, TIAR, ALS, FTLD, FXS/FXTAS, PEM/PSN

## Abstract

The timing, dosage and location of gene expression are fundamental determinants of brain architectural complexity. In neurons, this is, primarily, achieved by specific sets of trans-acting RNA-binding proteins (RBPs) and their associated factors that bind to specific cis elements throughout the RNA sequence to regulate splicing, polyadenylation, stability, transport and localized translation at both axons and dendrites. Not surprisingly, misregulation of RBP expression or disruption of its function due to mutations or sequestration into nuclear or cytoplasmic inclusions have been linked to the pathogenesis of several neuropsychiatric and neurodegenerative disorders such as fragile-X syndrome, autism spectrum disorders, spinal muscular atrophy, amyotrophic lateral sclerosis and frontotemporal dementia. This review discusses the roles of Pumilio, Staufen, IGF2BP, FMRP, Sam68, CPEB, NOVA, ELAVL, SMN, TDP43, FUS, TAF15, and TIA1/TIAR in RNA metabolism by analyzing their specific molecular and cellular function, the neurological symptoms associated with their perturbation, and their axodendritic transport/localization along with their target mRNAs as part of larger macromolecular complexes termed ribonucleoprotein (RNP) granules.

## 1. Introduction

**Figure 1:**
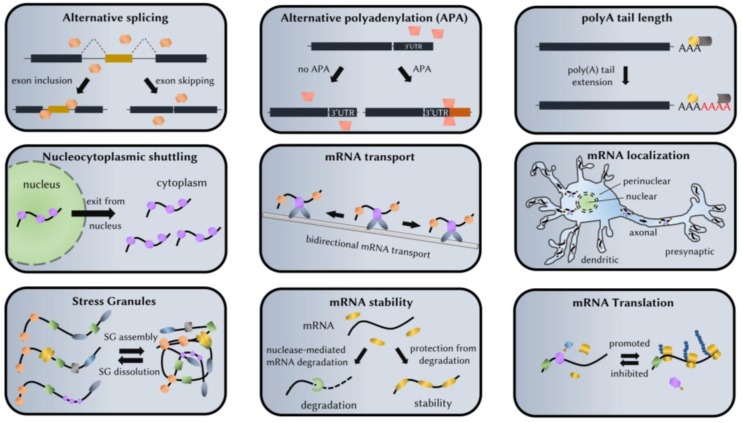
Roles of RNA-binding proteins in RNA processing.

The human brain contains some 86 billion neurons [1], each with a unique set of connections that run into thousands per neuron that constantly remodel during lifetime based on inputs received from neighboring neurons and non-neuronal cells. It is intuitive that, nowhere else in the body, the cells go to similar lengths in order to use the available strategies that diversify their functional repertoire from the finite number of genes. Thus, enhanced gene expression and alternative mRNA processing events that include splicing, polyadenylation, editing, stability, temporal silencing, targeted localization and translation (Figure 1) are all more prevalent in the brain as well as follow more distinctive patterns [2,3,4,5]. These events, collectively known as the ribonome, are mediated by RNA-binding proteins (RBPs) (reviewed in [6]), many of which are brain-restricted, each recognizing specific nucleotide sequences or structural motifs on target mRNAs.

Classical RBPs bind mRNA targets via conserved RNA-binding domains (RBDs) that are in most cases found in multiple copies (usually two or three) and/or in different combinations within the same protein. They are distinguished into four broad categories: the three single-stranded RNA recognition domains that include the RNA recognition motif (RRM) [7], the heterogeneous nuclear ribonucleoprotein (HNRNP) K-homology domain (KH) [8], and the zinc finger domain (Znf) [9]; and the double-stranded RNA-binding domain (dsRBD) that recognizes RNA double helical structures [10]. In general, each RBD has intermediate affinity and specificity for the RNA targets; however, strong and selective binding is obtained by the combinatorial use of adjoining RBDs. In addition, most RBPs possess intrinsically disordered regions, characterized by monotonous repetitions of distinct amino acids such as R/G, S/R, and K/R repeats that prevent folding into stable secondary or tertiary structures. These repeats contribute to both the specific and non-specific binding of RBPs to RNAs and mediate reversible interactions with other proteins, particularly other RBPs (reviewed in [11]).

During and following transcription, RBPs are thought to spontaneously couple to mRNA targets to facilitate processing of pre-mRNAs. Thereafter, many of these mRNA-bound RBPs, due to their low complexity domains, attract the same or different RBPs building larger ribonucleoprotein (RNP) granules around the mRNA target-scaffold. The interaction between bound RBPs and the mRNA ultimately determines the localization of the transcript and subsequent translation or degradation. RNP granules are highly dynamic in nature, assembling and dissembling depending on local environment. In extreme conditions of cellular stress, such as hypoxia, heat-shock, or oxidative stress, RNP granules come together and package into reversible membrane-less stress granules (SGs) which arrest translation in order to conserve energy and minimize stress-related damage (e.g., from the accumulation of misfolded proteins). This effect is mediated primarily by nucleating RBPs such as TIA1/TIAR, G3BP, eIF3 complex, and poly(A)-binding protein (PABP) (reviewed in [12]). Interestingly, many amyotrophical lateral sclerosis/frontotemporal dementia (ALS/FTD)-linked mutations on several RBPs (e.g., TDP43, FUS, TIA1) drive the formation of aberrant SGs both *in vitro* and in vivo suggesting that mRNA mislocalization and/or loss of RBP function may be a common feature of pathogenic mechanisms in neurodegeneration (discussed in [6]).

Several recent studies have revealed that as many as 2550 mRNAs are present in axons and dendrites [13] and that the mRNA localization is the primary determinant of local protein translation in these compartments [14,15,16]. Perhaps surprisingly, despite the huge number of localized mRNAs and the vastly smaller number of RBPs identified so far in axons and dendrites, transport of mRNA transcripts to final destinations is thought to occur singly, i.e., no two or more molecules of the same or different mRNA species occur in the same RNP granule [17,18].

Once RNP granules have assembled, active neuronal mRNA transport to axons and dendrites requires the association of RBPs—either directly or via protein adaptors—with the molecular motors kinesins (for + end movement) and dyneins (for − end movement) which provide fast bidirectional transport along the cell’s microtubule network [19,20,21,22]. Importantly, during the transport process, mRNAs are kept in translationally-repressed states until they reach their target destination. RBPs exploit different methods to block translation in RNP granules. Some recruit members of the 4E-BP family of proteins that bind eukaryotic translation initiation factor eIF4E and block translation during transit [23,24,25]. Some RBPs bind directly ribosomes and reversibly stall them while assembled on target mRNAs [26], whereas still others recruit de-adenylase proteins to shorten poly(A) tail length and prevent efficient binding of the cytosolic poly(A) binding proteins (PABPs) required for efficient translation initiation [27]. Once RNP transport granules have reached their final destination, release of mRNAs in axodendritic terminals and subsequent local translation requires, in part, the post-transcriptional modification of the bound RBP. These include (de)phosphorylation and methylation and are mediated by locally-hosted enzymes in response to intracellular signaling cascades [28,29,30,31].

In this review, we aim to link recent scientific findings on the basic molecular function of different RBPs with their role in neuronal homeostasis and neurological disease (summarized in Table 1). We elaborate on the role of each RBP in axodendritic transport and translation of target mRNAs and include survival motor neuron (SMN) protein in the analysis, as it is an important disease-associated co-partner in alternative splicing and RNP transport. We conclude by highlighting the recurrent themes observed from the deregulation of these proteins. 

## 2. Neurodevelopmental Defects

### 2.1. Pumilio

Pumilio (Pum) belongs to the evolutionary conserved Pumilio and FBF (PUF) family of RBPs comprised of two paralagous members in vertebrates (Pum1-2) and one in *Drosophila* (Pum) [32]. The characteristic feature of these proteins is their Pumilio homology domain (Pum-HD), which serves for binding to single-stranded RNA sequences with the consensus UGUANAUA [33,34,35]. PUM1 and PUM2 proteins share 83% identity, whereas their highly conserved Pum-HD is 91% identical [32]. Pum genes have widespread and largely overlapping tissue expression patterns, which suggest that they may have redundant or complementary function [32,36]. They primarily repress gene expression post-transcriptionally, via either direct interaction with the 5′ 7mG cap structure of mRNAs antagonizing the binding of translation initiation factor eIF4E or via binding to *eIF4E* mRNA lowering its expression [37,38,39]. Often in close association with *Drosophila* Nanos (NOS), another RBP that embraces Pum and mRNA to increase Pum’s RNA-binding affinity [35], Pum regulates stability and translation of mRNA molecules involved primarily in embryonic development and germline stem cell proliferation (reviewed in [40]).

Studies from invertebrate and vertebrate systems have identified Pum as important mediator of neurological processes. In *Drosophila*, Pum along with Staufen are involved in memory formation after olfactory learning [41]; and in conjunction with Nanos, Pum is essential for dendrite morphogenesis in peripheral neurons [42] and the regulation of sodium currents in motoneurons [43]. Knockout studies (KO) in mice have demonstrated that Pum1 haploinsufficiency (complete knockout dies at the pre-implantation stage [44]) causes progressive motor dysfunction and spinocerebellar ataxia type 1 (SCA1)-like neurodegeneration with motor impairment, primarily by increasing Ataxin1 (ATXN1) levels (a protein that accumulates in neurons and exerts neurotoxicity) [45]. Interestingly, in a recent report, the same research group identified human patients with Pum1 mutations that were associated with adult-onset ataxia, developmental delays and seizures [46]. Pum2 has been more widely studied than Pum1, and revealed several developmental stage-dependent effects in neuronal physiology. Neural-specific inactivation of Pum2 severely reduced the number of neural stem cells in postnatal dentate gyrus, increased perinatal apoptosis and impaired learning and memory [47]. *Pum2*-KO immature neurons demonstrated enhanced dendritic outgrowth and arborization, while *Pum2*-KO mature neurons exhibited abnormal neuronal morphology alongside a significant reduction of dendritic spines and increased elongated filopodia. Furthermore, an increase in excitatory synapse markers along dendritic shafts was recorded [48,49]. Pum2 expression has been associated with epilepsy, a condition characterized by extensive neuronal excitability and abnormal high levels of synchrony between neurons. In brain tissues of patients suffering from temporal lobe epilepsy (TLE), Pum2 levels are decreased. The same findings were also demonstrated in rats induced with a TLE experimental model [50] and in mice deficient of Pum2 [49]. Furthermore, *Pum2^XE772^*-homozygous mice expressing a Pum2 form that lacks the Pum-HD domain, exhibited behavioral and pathological abnormalities such as impaired spatial and memory capacities without major morphological defects in the brain [51].

Pum2 has also been implicated in the transport of mRNA transcripts. Pum2 tissue staining demonstrated small punctuate particles found in both the soma and the dendrites of fully polarized neurons indicating that it is involved in RNP transport to dendrites [38]. Furthermore, when overexpressed in neurons and fibroblasts, Pum2 induced the formation of SGs recruiting core components such as TIA1/TIAR, eIF4E, PABP, Staufen1 and Barentsz [38]. *Pum2* downregulation in neurons via RNA interference (RNAi) impeded with the formation of SGs during stress, suggesting that it is a core component of SGs. Interestingly, Pum2 remained excluded from processing bodies (P-bodies) under all experimental conditions [38]. One of Pum1/2’s negatively regulated mRNA targets in dendrites is *eIF4E*, an important translation initiation factor at synapses [39,48].

Collectively, Pum is a translation repressor involved in the early stages of neuronal development, whose lowered expression in pathological conditions drives neuronal hyper-excitability and epileptic seizures.

### 2.2. Staufen

Staufen (Stau) is a double-stranded RNA-binding protein. A single gene has been described in invertebrates and two paralogs termed *Stau1* and *Stau2* were described in vertebrates, [52,53,54,55]. *Stau1* and *Stau2* are approximately 60% identical [54]. *Stau1* is ubiquitously expressed [52], whereas *Stau2* expression is restricted to brain and heart [56]. Stau −1 and −2 reside primarily in the cytoplasm; however, both proteins display also nuclear localization, preferentially nucleolar [57].

Stau was originally identified for its role in the localization of mRNAs encoding cell fate determinant proteins essential for the anterior–posterior patterning of the *Drosophila* oocyte [55]. Subsequent work with Stau mutants in *Drosophila* revealed its role in brain development. Specifically, Stau was shown to mediate the asymmetric localization of *Prospero* mRNA, a transcription factor that suppresses stem cell fate, to the future ganglion mother cell (GMC) away from neuroblasts, thus promoting differentiation [58]. More recently, two studies in mice showed that Stau2 begins to polarize in mitotic cells during early neurogenesis where it localizes to the differentiating cell. Knockdown of *Stau2* with shRNAs yielded a significant increase in the number of differentiated daughter cells with a concomitant decrease in Pax6-positive radial glial cells reproducing earlier findings in *Drosophila* [59,60]. The role of Stau is not limited to early development but also in synaptic plasticity and memory formation in the mature nervous system. Using siRNA-mediated *Stau1* knockdown in hippocampal pyramidal neurons, Lebeau et al. reported impaired late form long-term potentiation (LTP) but not early-LTP or basal evoked synaptic transmission. Stau1 downregulation also decreased the amplitude and frequency of miniature excitatory postsynaptic currents, suggesting a role in homeostatic synaptic efficacy. At the cellular level, Stau1 downregulation shifted spine morphology from regular to elongated spines, without alterations in spine density [61,62]. Additionally, hippocampal neurons from mutant mice that express a truncated Stau1 protein lacking the functional RNA-binding domain 3 showed significantly reduced dendritic tree arborization and developed fewer synapses, along with deficits in locomotor activity [62]. Morphological defects in dendritic spines and decreased number of synapses were also demonstrated in hippocampal neurons lacking the expression of Stau2 [63]. These observations highlight the conserved role of Stau proteins in synaptic plasticity.

The number of mRNAs associated with Stau in the brain and cell lines run into several hundred; however, the steady-state levels of only a fraction of mRNAs is influenced by Stau2 downregulation in neurons [64,65,66]. In these cases, Stau −1 and −2 either enhanced translation or promoted staufen-mediated decay (SMD) via recruitment of Upf1 to 3′ untranslated region (UTR) of mRNA targets [67,68,69]. Staining of Stau revealed a punctuate somatodendritic distribution pattern in hippocampal neurons [70] in tight co-localization with RNA granules [71] with Stau1 and Stau2 displaying distinct granules [72,73]. Recruitment of Stau into granules as well as its subsequent transport to dendrites required involvement of microtubules [71,74]. Furthermore, the migration of Stau2 and of RNA species to the dendrites was parallel and dependent on neuronal activity [75]. Expression of a truncated Stau2 form that lacks the c-terminal portion required for dendritic targeting, restricted Stau expression to the cell bodies and reduced total dendritic RNA by 40%, while concomitantly increased somatic RNA content [76]. Furthermore, in the rat hippocampus, Stau2 co-purified with mRNAs localized in the neuronal processes but not cell bodies, further confirming Stau’s major involvement in dendritic mRNA localization [66].

Upon exposure to oxidative stress conditions, both Stau proteins are recruited into SGs and co-localized with TIAR and HuR. Of note, the redistribution of Stau proteins in SGs happened simultaneously with the presence of polyadenylated RNAs in SGs [73]. Interestingly, Stau1 overexpression impaired SG formation, whereas Stau1 knockdown impaired their dissolution [77].

Collectively, these data confirm the key role of Stau proteins on polarized mRNA localization during early neurogenesis and in somatodendritic RNP transport during synaptic plasticity.

### 2.3. Insulin-Like Growth Factor 2 mRNA-Binding Protein (IGF2BP) 

The insulin-like growth factor-2 mRNA-binding proteins 1, 2, and 3 (IGF2BP1, IGF2BP2, IGF2BP3) belong to the IGF2BP family of conserved RNA-binding pro-tumorigenic fetal proteins. Nomenclature is sometimes confusing due to the many synonyms used, which include VgLE binding and ER association (Vg1RBP/Vera) in *Xenopus*, IGF2 mRNA-binding protein (IMP) 1–3 in mammals, zipcode-binding protein (ZBP) 1 in chicken as well as function-based acronyms such as KOC and coding region determinant-binding protein (CRD-BP). There is approximately 56% amino acid sequence similarity between the three proteins with greater degree of homology seen within their RNA recognition motifs suggesting shared biochemical functions [78]. Unlike other RBPs, they are predominantly cytoplasmic and are presented in large 200–700 nm RNP granules [79]. Photoactivatable ribonucleoside-enhanced crosslinking and immunoprecipitation (PARCLIP) has revealed that IGF2BPs bind to CAUH (where H = A, U, or C) consensus recognition motif [80] while enhanced CLIP (eCLIP) to CA-rich motifs [81]. IGF2BP1 and IGF2BP2 were found to bind predominantly to 3′ UTRs of target mRNAs, whereas IGF2BP3 bound a higher portion of coding region [81]. IGF2BPs are primarily expressed during development showing overlapping expression in multiple tissues. However, unlike IGF2BP1 and IGF2BP3, IGF2BP2 expression is maintained in adult mouse tissues including brain [78].

Loss-of-function studies have identified IGF2BPs as essential modulators of cell growth and differentiation during development. IGF2BP1-KO mice are on average 40% smaller than wild-type littermates with proportional reduction in the size of all organs including brain. In addition, 50% die perinatally with the cause of death attributed primarily to gut defects [82]. Similarly, IGF2BP2-KO mice also displayed slightly smaller size than controls and had increased energy expenditure and decreased fat deposition [83]. With respect to neuronal function, ablation or overexpression of neuronal IGF2BP in *Drosophila* resulted in compromised central and peripheral synaptogenesis due to altered synaptic terminal growth [84]. In *Xenopus*, depletion of IGF2BP revealed its requirement for the migration of cells forming the roof plate of the neural tube and, subsequently, for neural crest migration [85]. Consistent with a role in early neuronal development, IGF2BP1 deletion in mice leads to neural stem cells (NSCs) becoming prematurely depleted in the dorsal telencephalon due to accelerated differentiation, resulting in reduced brain mass [86]. It was proposed that IGF2BP1 post-transcriptionally inhibited the expression of differentiation-associated genes while promoted the expression of self-renewal genes [86]. IGF2BP1 loss in human pluripotent stem cells (hPSCs) revealed a reduction in cell adhesion and an increase in cell death [81]. Consistent with IGF2BP2’s high expression in the developing brain, overexpression of IGF2BP2 increased the neurogenic potential and suppressed astrocytic differentiation of late-stage neural precursor cells (NPCs), whereas knockdown of IGF2BP2 promoted astrocytic differentiation and reduced the neurogenic potential of early-stage neocortical NPCs without overtly affecting cell proliferation [87].

Numerous studies have revealed IGF2BPs role in controlling the transport, localization and expression of target mRNAs. Outside the nervous system, IGF2BPs are found in the form of RNP granules localized around the nucleus and in cell protrusions. In neurons, IGF2BP RNP granules localize in dendrites and growth cones [88,89]. These RNP granules are transported along microtubules towards the leading edge [79]. Apart from IGF2BPs, mRNAs and associated RBPs, these RNP granules also contain the small ribosomal subunit 40S, poly(A)-binding proteins, CBP80 (NCBP1, a 5′ cap binding protein) and proteins of the exon–junction complex (EJC) but lack eIF4E, eIF4G and large ribosomal subunit 60S, suggesting that the constituent mRNA transcripts are not translated while transported and that once released, translation is fine-tuned by earlier splicing-dependent events [90,91]. Ser181 phosphorylation by mTORC2 pathway [28] as well as binding to SMN [92] are required for IGF2BP1 mobility and dendritic localization. The best-characterized mRNA constituent of IGF2BP RNP granules is *Actb*. IGF2BP1 binds *Actb* 3′ UTR and transports it to dendrites and growing axons. Once there, in response to trophic factors, IGF2BP1 is phosphorylated at Tyr396 by SRC kinase and releases *Actb* mRNA for translation [93]. Apart from neurotrophic factors, the transport, localization and local translation of *Actb* mRNA is also enhanced by *N*-methyl-d-aspartate (NMDA) receptor activity [94]. This regulation ultimately drives proper axonal growth cone navigation and dendritic branching [95,96,97,98,99,100]. IGF2BPs have also been implicated in the nerve regeneration capacity of adult neurons. IGF2BP1 +/− neurons displayed axonal growth deficits after transection or crush injury of peripheral dorsal root ganglion (DRG) nerves [100].

Collectively, these findings identify IGF2BPs as key regulators of neuronal development via control of NSC proliferation, neuronal cell migration and specification, as well as neurite outgrowth by spatiotemporal fine-tuning protein synthesis in both axons and dendrites as demonstrated for *Actb* mRNA.

### 2.4. Fragile-X Mental Retardation Protein (FMRP)

The fragile-X mental retardation protein (FMRP), encoded by the FMR1 gene, is an RNA binding protein predominantly expressed in the brain (particularly in neurons) and gonads [101,102,103,104]. Although the bulk of FMRP resides in the cytoplasm, a portion of FMRP is detected in the nucleus and within nuclear pores [105]. FMRP is a multi-domain protein harboring among others two KH domains and a single Arg-Gly-Gly-rich (RGG-type) box for RNA and protein binding [103,106] of which KH2 is perhaps the most critical for function [101,103,107,108,109]. In marked contrast to other RBPs, cross-linking immunoprecipitation high-throughput sequencing (HITS-CLIP) revealed that FMRP binds most frequently to the coding region of mRNAs as opposed to 5′-, 3′-UTRs and introns with a preference for TGGA sequences among other motifs [26,110,111].

FMRP is responsible for the fragile-X syndrome (FXS), the first neurological disease associated with a dysfunction in RNA metabolism explicitly. FXS is the most common form of inherited mild to severe mental retardation and the most frequent monogenic cause of autism spectrum disorder (ASD) [112]. It is caused by a CGG triplet repeat expansion within the 5′ UTR of the FMR1 gene resulting in an abolished, or greatly diminished, expression of FMRP. Normally, there are between 5 and 40 repeats. Individuals with 55 to 200 repeats have a near normal intellect but those with greater than 200 CGG repeats display the full spectrum of the disease [113,114,115]. Mechanistically, the CGG expansion transcriptionally silences *FMR1* expression by promoting hypermethylation of the gene locus [116] and by the complementary binding of the trinucleotide repeat *Fmr1* mRNA to the promoter region [117]. Several animal models (flies, mice, rat, zebrafish) have been developed over the years to study FXS (reviewed in [118] and [119]). Most employ knockout of *Fmr1* and can recapitulate many of the FXS symptoms such as deficits in learning and memory, hyperactivity, altered morphology of dendritic spines, and ASD-like pattern of social interaction [120,121,122,123,124,125]. Interestingly, while FXS patients exhibit mild neuronal loss in a variety of neuronal tissues [126], experiments preformed in *Drosophila* and mice showed only reduced programmed cell death during early development with retention of normally transiently-lived cell populations, an observation which agrees with the hyperexcitability symptoms of FXS [127,128]. To reconcile these differences, one can speculate that the altered neuronal dynamics during lifetime result in eventual neuronal degeneration in long-lived organisms, which is not applicable for short-lived animals.

The contribution of FMRP in axodendritic morphology has been investigated in detail. Human brain autopsy material has shown that FXS patients demonstrated increased ratio of long over short dendritic spines in comparison to healthy subjects in the cortex. In addition, FXS neurons exhibited significantly less dendritic spines with a mature morphology and more with less mature-type morphology in the same area [129]. Morphological studies done in KO mice and null-mutant *Drosophilas* have yielded similar findings to human autopsies. Dendritic spines were found to be denser, smaller, had higher turnover rates, and showed greater developmental delay in the transition from immature to mature spine subtypes [130,131,132,133].

At the molecular level, FMRP has been proven an effective repressor of target mRNA expression. Its inhibitory effect is achieved by different means including ribosomal stalling [26] and trapping of target mRNAs in large cytoplasmic granules [134]. With respect to polyribosome stalling, it is yet not clear whether this involves direct binding of FMRP to ribosomes or requires other protein intermediates [135]; however, the KH2 domain is essential for this interaction [136,137]. FMRP, additionally, has been shown to prevent the interaction of eukaryotic initiation factors eIF4E and eIF4G, which are required for ribosome recruitment. This inhibitory effect is mediated, at least in part, via its binding partner cytoplasmic FMR1-interacting protein (CYFIP) 1, a 4E-BP translation inhibitor. At synapses, BDNF or DHPG stimulation of neurons causes CYFIP1 to dissociate from eIF4E, thereby allowing protein synthesis to proceed [138]. One other way that FMRP was shown to regulate the translation silencing of target mRNAs is through its association with DICER1 and Argonaute 2 (AGO2), center-piece components of the RNA-Induced Silencing Complex (RISC) [139]. Several reports have indicated that FMRP binding to target mRNA contributes to the ability of the miRNA-RISC complex to recognize the target mRNA and repress its expression [140,141,142]. Post-translational modifications of FMRP regulate its functions. Sumoylation [143], methylation [144] and dephosphorylation by PP2A [29,140] blocked the translation suppression effect of FMRP. Phosphorylation at Ser499 [145,146,147] on the other hand promoted the recruitment of RISC complex on FMRP-bound mRNAs [140] and increased the association of FMRP with stalled polyribosomes [148]. Although the bulk of research has aimed at the synaptic targets of FMRP some 13% of them, identified by HITS-CLIP, encode for transcription factors and chromatin modifiers [26] suggesting that FMRP’s role may also be extended to the nucleus. Indeed, Korb et al. revealed that *Fmr1* KO mice show histone modifications associated with open chromatin resulting in increased expression of many critically synaptic genes [149]. This provided important clues in understanding FXS and revealed an unexpected link between direct translation and indirect transcriptional enhancement following FMRP loss. More recently, novel roles have been assigned to FMRP based on its nuclear localization. FMRP was found to co-precipitate in an RNA-dependent fashion with an alternative splicing-associated protein, the RNA-binding protein 14 (RBM14). FMRP promoted RBM14 binding to its mRNA targets. Knockdown of either FMRP or RBM14 altered the relative skipping/inclusion ratio of targeted exons including those of Protrudin (ZFYVE27, kinesin adapter) and microtubule-associated protein TAU [150]. FMRP was also shown to bind chromatin through its tandem Tudor domain and participate in DNA damage response (DDR) in a chromatin-binding-dependent manner. DDR is critical for long-lived cells, such as neurons, since the accumulation of mutations could increase the chances of dysfunction initially at cellular and later at network level. *Fmr1* fly mutants were consistently unable to recover from genotoxic stresses compared to wild-type controls [151]. An unexpected function of FMRP in modulating the activity of adenosine deaminase acting on double-stranded RNA-specific adenosine deaminase (ADAR) enzymes has also been reported in *Drosophila*, Zebrafish and mice KOs. ADARs bind double-stranded RNA, generated by the hybridization of complementary exon and intron sequences in the pre-mRNA of specific transcripts, to convert adenosine A to inosine I by direct deamination (reviewed in [152]). This RNA editing event is read by the ribosomes as a guanosine (G) and is thought to contribute to the diversification of the mRNA pool. The process has been described primarily for neuronal mRNAs and changes in editing patterns have been associated with psychiatric disorders (reviewed in [153]). Accordingly, FMRP was shown to physically and biochemically interact with ADARs. Animal KOs of FMRP displayed mostly an increase in the editing levels of brain specific mRNAs, indicating that FMRP acts as an inhibitor of editing activity [154,155,156]. Last but not least, FMRP was shown to directly bind ion channels to modulate Ca^+2^ signaling and neurotransmitter release. These included voltage-gated [157] and Ca^+2^-activated/voltage-gated potassium channels [158].

FMRP’s role in mRNA transport and presynaptic terminals has also been extensively researched. FMRP has been observed in motile RNP granules that localize in the growth cone and distal segment of the axon with pockets of higher concentrations appearing intermittently [159]. *Map1b* mRNA, a target of FMRP, was co-localized in the growth cone and *Fmr1* KO neurons displayed reduced mobility of growth cones [159]. FMRP granules contained ribosomes (albeit in a different subdomain), polyadenylated RNAs and surprisingly only a small subset of the known FMRP target mRNAs indicating that FMRP is not involved in the transport of its targets but mainly in translation regulation. Nevertheless, for a subset of mRNAs present in those granules, FMRP expression was important for both subcellular distribution and abundance [160]. Additionally, FMRP has been observed to co-localize with Stau in RNP granules that contained many of the P-body components such as the RNA-degrading enzymes Dcp1p and Xrn1p, miRISC component Ago, nonsense-mediated decay (NMD) surveillance protein Upf1p, and general translational repressors such as Dhh1p, once again indicating that it is solidly involved in the repression of mRNA expression [161,162]. Finally, studies in mice have revealed that FMRP acts as an adaptor of kinesin-1 for microtubule-based transport of translationally dormant mRNPs [163,164]. These FMRP granules persist in adulthood in both rodents and humans indicating preserved function throughout life [165].

Overall, FMRP has got pleiotropic effects ranging from the well-established role in translation repression of mRNAs in the cytoplasm to transcriptional inactivation via specific actions on chromatin modifiers, DDR to genotoxic stress and inhibition of RNA editing in the nucleus. Loss-of-function mutations result in the development of FXS presented by alterations in dendritic spine numbers and maturation.

### 2.5. Src-Associated Substrate in Mitosis of 68 kDa (Sam68)

Src-associated substrate in mitosis of 68 kDa (Sam68), also known as KHDRBS1 (KH domain containing, RNA binding, signal transduction associated 1) is an RBP that binds nonspecifically to poly(U) RNA and specifically to the high-affinity binding sequences UAAA, in vitro and in vivo [166,167,168]. Like other RBPs, it is involved in alternative splicing and transport but, unlike most other RBPs that act as repressors of translation, Sam68 promotes translation of its targets via polyribosome recruitment [169,170]. Moreover, it is the prototypic member of the STAR (Signal Transduction Activator of RNA) family of proteins that links signaling pathways to various aspects of post-transcriptional regulation and processing of RNAs. It serves as a docking scaffold in response to the activation of several membrane-bound receptors and frequently undergoes post-transcriptional modifications that modulate its localization, RNA affinity and interaction with signaling proteins (reviewed in [171]). It is ubiquitously expressed and high levels are detected in neuronal and glial cells in both brain and spinal cord in both white and gray matter [172]. Strong immunoreactivity is also detected in neurogenic areas of the neocortex where it supports the self-renewing potential of neural progenitor cells (NPCs) [173]. Sam68-KO mice are presented with constricted pools of proliferating NPCs by hastening their cell cycle exit and differentiation into post-mitotic neurons [173]. While in unstimulated conditions Sam68 is predominantly nuclear, in response to stimulatory signals, half of its amount translocates to the neuronal soma and dendrites, in a microtubule-dependent process [174]. Following neuronal activity, Sam68 regulates the alternative splicing of neurexins (*Nrxns*), an important class of cell adhesion molecules required for the assembly of presynaptic terminals and hence synaptic function. Sam68-KO mice display severe perturbation of Nrxn-1 splice variants and stimulation of cerebellar or cerebral neurons with kainic acid was significantly attenuated [175]. These mice have significant motor coordination deficits [175]. In a contusive spinal cord injury (SCI) model, Sam68 levels were significantly increased. Immunofluorescence staining revealed that Sam68 expression was co-localized with NeuN (RBFOX3) and Caspase-3 (CASP3) indicating neuronal apoptosis and with PCNA and GFAP indicating reactive astrogliosis. Both effects were hampered following knockdown of Sam68 and were accompanied with reduced cyclin-D1 levels, indicating that Sam68 is promoting neurodegeneration following injury [172]. Sam68 in conjunction with HNRNPA1 has also been shown to influence 5′ splicing of *Bcl-x* (*Bcl2l1*) mRNA regulating prosurvival and apoptotic pathways. Neutralization of Sam68 by RNAi caused accumulation of antiapoptoticBcl-x(L), whereas its upregulation increased the levels of proapoptoticBcl-x(s) [176]. Moreover, Sam68 is required in the tumor necrosis factor (TNF) apoptotic signaling pathway, where it acts as a signaling adaptor for both the membrane-associated complex I and cytoplasmic complex II, modulating nuclear factor kappa B(NF-κB) activation and, thereafter, apoptosis [177].

There are several indications pointing towards a role of Sam68 in RNA transport and local translation at synapses. Sam68 cosedimented with polysomes from synaptosomal fractions and Sam68 immunoreactivity, analyzed with electron microscopy, was associated with dendritic microtubules, endoplasmic reticulum, and free polyribosomes, at times close to synapses [178]. Following neuronal stimulation, dendritic Sam68 is present in the form of granules, 26% of which are co-localized with ethidium bromide-stained RNA clusters, pointing towards RNP granule formation. Most of the granules were stationary, but a few migrated in either a retrograde or anterograde direction [174]. Sam68 has also been shown to bind *β-actin* (*Actb*) mRNA, an integral cytoskeletal component of dendritic spines. Consequently, Sam68-KO mice have reduced levels of *Actb* mRNA associated with synaptic polysomes and lower levels of synaptic ACTB protein, indicating that Sam68 promotes the translation of *Actb* mRNA at synapses. In addition, neurons from Sam68-KO mice possessed fewer dendritic spines [170]. In response to oxidative stress, Sam68 is recruited to SGs and co-localizes with TIA1; nevertheless, Sam68 knockdown has no effects on SG assembly, indicating that Sam68 is not a constitutive component of SGs [179].

Besides the proapoptotic role of Sam68 in spinal cord injury, Sam68 has also been implicated in the onset of two human neurodegenerative diseases. Recent studies have demonstrated that Sam68 at first, and two other RBPs, the MBNL1 and HNRNPG later, are sequestered by expanded CGG repeats (55–200) on the *Fmr1* mRNA [180], causing Fragile-X-associated tremor/ataxia syndrome (FXTAS). This late-onset disorder is characterized by action tremor, gait ataxia and executive cognition dysfunction and is likely caused by titration of RBPs away from their physiological targets. Consequently, Sam68-responsive splicing is altered in FXTAS patients [180]. In the neurodegenerative disorder spinal muscular atrophy (SMA), Sam68 in conjunction with hnRNPA1 has been shown to be an important regulator of *Smn2* pre-mRNA alternative splicing, acting as a splicing repressor of exon 7 inclusion [181]. The result is the production of a truncated highly unstable SMN2 protein that cannot support the survival and function of spinal motor neurons when *Smn1*, the original gene that during human evolution was duplicated [182], is mutated or deleted. Accordingly, SAM68 KO mice promote *Smn2* splicing and expression in SMAΔ7 mice (that carry a homozygous deletion of the mouse *Smn* gene), partially rescuing SMA-related defects in motor neurons and skeletal muscles [183]. In a separate study, Narcis et al. revealed that in SMAΔ7 mice there is also abnormal nuclear accumulation of Sam68 which was accompanied by changes in the alternative splicing of the Sam68-dependent *Bcl-x* and *Nrxn1* genes, as well as changes in the relative accumulation of the intron-containing *Chat*, *Chodl*, *Myh9* and *Myh14* mRNAs, which are all important for motor neuron functions [184].

In conclusion, Sam68 is a pleiotropic protein that, besides being a signaling adaptor, it displays strong RBP-related functions. With respect to neuronal function, it is involved in NSC proliferation and in developing neurons in dendritic arborization and synaptic plasticity via regulation of alternative splicing, transport and translation.

### 2.6. Cytoplasmic Polyadenylation Element Binding (CPEB)

CPEB1 belongs to the cytoplasmic polyadenylation element binding (CPEB) family of proteins comprised of four paralagous members in vertebrates (CPEB1-4) and two in invertebrates. All members are widely expressed with overlapping pattern [185,186,187]. CPEB1, referred to as CPEB, binds to cytoplasmic polyadenylation elements (CPEs, UUUAU or UUUUAAU consensus sequence) found in the 3′ UTR of target mRNAs and modulates poly(A) tail length [188]. Members CEPB2-4 have weak affinity for CPEs and perform different functions [187,189]. The mechanism of action of CPEB1 was originally delineated in *Xenopus* oocytes [190], but, more recently, most of the auxiliary components have been identified in neuronal dendrites, too [191]. Cytoplasmic polyadenylation begins in the nucleus, where mRNAs with CPEs are bound by CPEB and CPSF before being exported to the cytoplasm. There, the RNP complex is joined by auxiliary proteins poly(A) polymerase Gld2, poly(A)-specific ribonuclease (PARN), Symplekin (SYMPK, an assembly factor), Maskin (*Xenopus* homolog to TACC-family of microtubule-interacting proteins), eIF4E, and ePAB (PABPC1L, poly(A) binding protein). PARN activity predominates and keeps the poly(A) tail shortened down to 20–40 nucleotides [27,192]. Stimuli that promote CPEB phosphorylation lead to the expulsion of PARN from the RNP complex and allow GLD2 to polyadenylate the bound RNA. As a result, ePAB binds the newly elongated poly(A) tail as well as eIF4G and displace Maskin from eIF4E allowing the initiation of translation [193,194]. Phosphorylation of CPEB1 in dendrites is mediated by aurora kinase A (AURKA) and/or calcium/calmodulin-dependent protein kinase type II alpha (CAMK2A) [191,195,196,197,198]. Apart from its role in cytoplasmic polyadenylation, nuclear CPEB is also involved in alternative polyadenylation [199].

CPEB is highly enriched at post-synaptic densities (PSDs) in dendrites, indicating that it is required for local translation and proper synaptic function [196,200]. Accordingly, *Cpeb1*-KO mice are presented with reduced theta burst-induced LTP in hippocampal neurons, a form of synaptic plasticity [201,202] and show a remarkable inability to extinguish memories (as new memories are consolidated) [203]. In addition, CPEB mice mutated at phosphorylation sites T171 and S177 in cerebellar Purkinje neurons display significant impairment of motor coordination and motor learning delay, reinforcing the overall importance of CPEB1 for synaptic function [204].

CPEB binds to and regulates expression of many transcripts involved in plasticity including the *Nr2a* subunit of the NMDA receptor, the *Glua1* and *Glua2* subunits of the α-amino-3-hydroxy-5-methyl-4-isoxazolepropionic acid (AMPA) receptor, *Camk2a*, and tissue plasminogen activator (tPA) [191,205,206,207,208,209]. Interestingly, *Camk2a’s* 3′ UTR (a protein of paramount importance for LTP) [210] seems to perpetuate a positive feedback loop with CPEB where CAMK2A phosphorylation of CPEB leads to reduced inhibition of CPEB-regulated *Camk2a* mRNA translation [195].

Regarding neuronal morphology, CPEB KO mice are presented with impaired mitochondrial function that is thought to affect dendritic branching [211] (reviewed in [212]). Similarly, knockdown of the protein using morpholinos in *Xenopus* tectal neurons resulted in reduced dendritic growth [213] and using a mutant form of the protein which is not affected by phosphorylation resulted in reduced dendritic spine number and length in Purkinje cells [204].

CPEB is also involved in mRNA transport. Huang et al. showed that CPEB1 associates with kinesin and dynein motors to bidirectionally transport mRNAs such as *Camk2a* and microtubule-associated protein 2 (*Map2*) to dendrites [214]. In neurons derived from CPEB KO mice, the dendritic transport of a CPE-containing reporter RNA was reduced [214]. CPE-containing mRNAs were transported to dendrites in a translationally dormant form, but activated at synapses in response to NMDA receptor stimulation [214]. CPEB has been observed in P-bodies [215] as well as in SGs following cellular stress; moreover, transient CPEB1 expression induces the assembly of SGs [216].

Overall, CPEB is an important mediator of cytoplasmic polyadenylation and therefore translation. It is involved in dendritic transport and local translation of mRNAs in dendrites contributing to synaptic plasticity.

## 3. Paraneoplastic Syndromes

### 3.1. Neuro-Oncological Ventral Antigen ( NOVA)

NOVA (neuro-oncological ventral antigen) family proteins contain two neuron-specific RBPs, NOVA1 and NOVA2. NOVA1 protein was originally identified as a target antigen in sera from patients with an autoimmune syndrome known as paraneoplastic opsoclonus myoclonus ataxia (POMA), a type of paraneoplastic neurodegeneration (PND) [217,218] characterized by increased motor movements sometimes leading to encephalopathy [219,220]. The two proteins display reciprocal expression in post-mitotic neurons [221], with NOVA1 being expressed in the hindbrain and ventral spinal cord, while NOVA2 is restricted in neocortex [217,218,222]. NOVA proteins contain three KH domains, with the KH3 domain being essential for binding to RNA species [223,224]. They shuttle between the nucleus and the cytoplasm with ~50% of NOVA proteins residing in the somatodendritic compartment [222,223]. NOVA proteins bind to YCAY (where Y is either a C or U) sequences on pre-mRNAs and regulate alternative splicing [223,224] including of their own mRNA [225,226].

There are several indications that NOVA proteins have an essential role in neuronal survival and function. *Nova1* KO mice are born indistinguishable from their littermates but die after 2–3 weeks from profound motor failure that correlates with apoptotic death of motor neurons in the spinal cord and brainstem [225]. Interestingly, POMA patients are also presented with neuronal cell death in the brainstem and spinal cord where NOVA1 is normally expressed [225]. Similarly, *Nova2* KO mice die a couple of weeks after birth and are characterized by aberrant migration of cortical and Purkinje neurons, whereas the NPC fate remains intact [221]. NOVA double-KO mice are born alive but are not motile and die immediately after birth due to lack of functional motor innervation of the lungs [227]. Furthermore, in *Nova2* KO hippocampal neurons, LTP is lost following external activation while basal synaptic transmission remains intact [228] indicating that NOVA2 is an important regulator of synaptic activity [229].

NOVA proteins regulate more than 700 alternative splicing events in vivo [230,231,232]. Similar to other RBPs, they promote the exclusion of alternatively-spliced exons by binding further upstream of them while their proximal downstream binding to introns promotes their inclusion [233,234]. Interestingly, some 230 transcripts showing changes in abundance in *Nova*-KO mice had NOVA sites mediating splicing of cryptic exons that ultimately triggered NMD. Most of these NOVA targets encoded synaptic proteins, including several implicated in familial epilepsy [235,236]. Further detailed analysis of knockouts revealed that NOVA2, but not NOVA1, uniquely regulated alternative splicing events of a series of axon guidance-related genes during cortical development [231,237]. Correspondingly, axonal pathfinding defects were specific to NOVA2 deficiency: *Nova2*-KO, but not *Nova1*-KO mice had agenesis of the corpus callosum and axonal outgrowth defects specific to ventral motoneuron axons and efferent innervation of the cochlea [231]. A high percentage of NOVA binding sites were also mapped near poly(A) sites in the 3′ UTRs of target mRNAs suggesting a role in alternative polyadenylation [234].

NOVA proteins have also been implicated in mRNA transport of several channels. Immunofluoresence and electron microscopy (EM) analysis of spinal cord motor neurons revealed that NOVA co-localizes with *GlyRα2* (*Glra2*) at PSDs. *GlyRα*2 is also a splicing target of NOVA in the nucleus [238]. Furthermore, binding of NOVA to the 3′ UTR of the *Girk2* mRNA directs the localization of a Girk2 reporter to neuronal processes [222]. 

Collectively, NOVA proteins are important mediators of alternative splicing and axodendritic mRNA transport and henceforth synaptic plasticity in neurons.

### 3.2. Embryonic Lethal/Abnormal Vision-Like (Hu/ELAVL) 

The Hu/ELAV-like proteins (HuR (ELAVL1, also known as HuA), HuB (ELAVL2, also known as Hel-N1), HuC (ELAVL3), and HuD (ELAVL4)) are mammalian homologs of the *Drosophila* embryonic lethal abnormal vision (ELAV) RNA binding protein, whose deletion—as the name suggests—was found to be lethal for flies [239]. HU proteins are 70% homologous at the protein level and contain three RRMs [240]. All four proteins bind U-rich sequences interspersed with Gs and secondarily with as [241,242]. HuR is ubiquitously expressed while HuB, HuC, and HuD are neuronally-enriched although HuB is also expressed in the gonads [243]. Subcellular localizations are somewhat different between the HU proteins: HuR is mainly nuclear and shuttles to the cytosol [244,245], HuC is present in both nuclear and cytosolic fraction and HuB and HuD are primarily cytosolic [246,247,248,249]. Each neuronal *Hu* displays a characteristic expression pattern during development: HuB is expressed in early post-mitotic neurons in the outer layer of the ventricular zone, and less in the intermediate zone and cortical plate. HuD is predominantly expressed in the intermediate layer and less in the ventricular zone and cortical plate, while HuC is only expressed in the cortical plate [240]. In adulthood, all neurons express from one to all *Hu* mRNAs depending on neuronal type.

HU proteins are important for neuronal development and plasticity illustrated primarily by loss-of-function studies. To explore the neuronal function of the otherwise ubiquitously-expressed HuR, tamoxifen-inducible, neuron-specific *HuR*-deficient mice were generated. These mice developed motor neuron disease characterized by poor balance, decreased movement and decreased strength. Immunostaining of the brain and cervical spinal cord revealed strong cleaved Caspase-3 expression in pyramidal and motor neurons of *HuR*-deficient mice. In addition, enriched Gene Ontology (GO) terms in the brain tissues of neuron-specific *HuR*-deficient mice were largely related to inflammation and showed similar patterns to those observed in ALS. Interestingly, neuronal *HuR* deficiency resulted in the redistribution of TDP43 to cytosolic granules, a typical feature of ALS [250]. *HuB*-KO mice have not been described yet. A study of *HuC*-KO mice revealed significant defects on the rotarod test exemplified by slowly progressive motor deficits leading to severe cerebellar ataxia [251,252]. Axons of *HuC*-KO Purkinje cells were swollen (spheroid formation), followed by the disruption of synaptic formation by axonal terminals. Deficit in anterograde axonal transport, in part due to deficits in KIF3A/C kinesins and abnormalities in neuronal polarity with proteins such as the somatodendritic MAP2 relocated in axonal terminals was observed in *HuC*-KO Purkinje cells [252]. These mice also displayed spontaneous epileptic seizure activity as a result of reduced glutamate expression [241] and impaired spatial learning [251]. *HuD*-KO mice exhibited, similar to HuC-KO mice, behavioral abnormalities and deficits, such as impaired spatial learning, lower levels of anxiety and activity, and predisposition towards auditory-induced seizures often resulting in death [253]. On the other hand, aberrant acquisition and retention of memories was observed in transgenic mice overexpressing HuD [254]. Furthermore, *HuD*-KO mice displayed an abnormal hind-limb clasping, which is often associated with cortical deficits as well as poor rotarod performance, suggesting motor defects [255]. At the cellular level, *HuD*-KO mice had the number of differentiating quiescent cells in the embryonic cerebral wall decreased and the number of slowly dividing stem cells in the adult subventricular zone increased, indicating that HuD is required for the exit of neural stem cells from cell cycle and for differentiation. *HuD*-KO mice also revealed a transient impairment in the neurite extension of cranial nerves during early embryonic development [255]. Additional studies revealed that *HuD* deficient motor [256] and hippocampal/neocortex neurons [253,257] exhibited reduced axonal and dendritic complexity. Despite the fact that both *HuC*- and *HuD*- KO mice do not display any gross anatomical variations, *HuC/D* double-KOs die just after birth, indicating that there is functional redundancy between them [241].

In humans, HU proteins have been linked to the paraneoplastic encephalomyelopathy/paraneoplastic sensory neuropathy (PEM/PSN) syndrome or simply anti-Hu syndrome. Patients with this syndrome are presented with sensory neuropathy, cerebellar ataxia, limbic/brainstem encephalitis, myelitis, short-term memory loss, epileptic seizures, and intestinal pseudo-obstruction. This syndrome is the outcome of an immune response to neuronal HU proteins that are ectopically expressed in certain tumors such as small-cell lung carcinoma (85% of cases) and neuroblastomas. These autoimmune responses involve the production of antibodies that cross the blood–brain barrier and injure neurons in a yet poorly understood manner (reviewed in [258]).

The wider role of HU proteins in neurological diseases is beginning to be elucidated. A polymorphism (rs10491817) in the first intron of HuB has been associated with schizophrenia, particularly in Asian populations [259]. A genome-wide association study (GWAS) and linkage analysis implicated HuD as a Parkinson’s disease (PD) susceptibility gene [260,261]. In the hippocampus of AD patients, neuronal HU protein levels decrease as a function of clinical dementia progression, with nuclear HU protein levels inversely correlated with αβ1–42 content [262]. The latter is not unexpected since HuD has been shown to regulate amyloid precursor protein (APP), beta-secretase 1 (BACE1) and antisense BACE1 levels [263] while HuB/C/D the neuron-specific alternative splicing of APP [264].

HU proteins are great multi-tasking proteins regulating different aspects of RNA metabolism in both cytoplasmic and nuclear compartments. In the nucleus, HU proteins participate in alternative spicing, stability and polyadenylation. High-throughput methodologies have revealed that as much as 30% of HU binding sites are found in introns [241,242,265]. Consequently, HU proteins have been shown to bind RNA pol II, histone deacetylase (HDAC) II and nascent pre-mRNAs, co-transcriptionally to modulate the speed of transcription and, thus, the inclusion of certain exons [266]. They also directly regulate alternative spicing of targets [267] or indirectly by enhancing the mRNA stability and translation of other splicing factors such as NOVA1 [268]. HU proteins, also, regulate alternative polyadenylation by interfering with cleavage stimulation factor (CSTF) 64 (CSTF2) binding to sites containing U-rich sequences near the polyadenylation sites [269]. In the cytoplasm, HU proteins enhance expression of target transcripts by either stabilizing mRNAs via 3′ UTR binding often antagonizing the action of destabilizing RBPs such as AUF1 [270,271,272,273,274], or competing with miRNAs [275,276,277] and/or promoting the translation of mRNAs by interacting with both the cap-binding protein eIF4A and poly(A) tail [264,277].

HU proteins have been linked to transport and localization of neuronal mRNAs including *Bdnf*, *Gap43*, *Tau*, *Neuritin/cpg15* (*Nrn1*), *Kv.1.1* (*Kcna1*) and *Camk2a* [256,278,279,280,281,282,283]. They display granular distribution in the axons and growth cones which, apart from bound mRNAs, contain dimers or trimmers of HU [284], other RBPs such as IMP1 and NOVA1, SMN, kinesin motor KIF3A, microtubule-associated MAP1B, and eIF4E [268,278,285,286,287,288].

Together, extensive evidence demonstrates that ELAVLs are important mediators of neuronal differentiation and synaptic plasticity, exerting their effects via modulation of all aspects of RNA metabolism in both the nucleus and axodendritic compartments.

## 4. Early-Onset Neurodegeneration

### Survival Motor Neuron (SMN)

Survival motor neuron (SMN) is a ubiquitously expressed and developmentally regulated gene [289]. The human genome contains a SMN1 orthologous gene, SMN2 (different in only five nucleotides), which primarily produces a truncated unstable protein due to exon 7 skipping (SMNΔ7), with only 10% of transcripts resulting in full-length functional SMN [290]. SMN is a multifactorial protein found in both nucleus and cytoplasm. It is, by now, well recognized that SMN associates with itself and at least eight additional proteins (GEMINs2–8 and UNRIP) to form a macromolecular complex referred to as the SMN complex. The SMN complex interacts with and assists in the assembly of Sm proteins and snRNAs into spliceosomal small nuclear ribonucleoproteins (snRNPs) (however, without being part of it), which are the essential components of the pre-mRNA splicing machinery catalyzing the excision of introns from mRNA precursors in the nucleus (reviewed in [291]). Studies employing the deletion of the *Smn1* gene revealed widespread intron retention, particularly of minor U12 introns [292,293,294] confirming the earlier biochemical studies on SMN1’s pivotal role in spliceosomal biogenesis.

In 1995, Lefebvre et al. discovered that mutations (deletions) in the *SMN1* gene cause spinal muscular atrophy (SMA), an autosomal recessive infantile neuromuscular disease where specific degeneration in spinal motor neurons takes place [295]. SMA occurs in approximately 1 in 11,000 newborns, and represents the most common hereditary disease-causing childhood death to date [296]. Clinically, the disease is manifested by profound muscle weakness, hypotonia, and trunk paralysis. The *SMN2* gene is a modifier of the SMA phenotype in that higher copy numbers of *SMN2* (the *SMN1/2* locus is highly unstable and variable) is associated with less severe clinical representation [297,298]. Antisense therapy is currently the best option for treating SMA. Several antisense oligonucleotide (ASOs) chemistries have been proven successful over the years in blocking the intronic splicing silencer site ISS-N1 on intron 7 of *SMN2*, driving the inclusion of exon 7 and the recovery of SMN protein expression from *SMN2* gene [299,300,301,302]. In December 2016, Nusinersen (the commercial name is Spinraza, by Ionis/Biogen) a 18-mer 2′-*O*-methoxyethyl phosphorothioate (2′MOE) modified oligo became the first drug to be approved by the FDA for the treatment of SMA [303].

Similarly to human, mice with homozygous *Smn* disruption (mouse genome contains only one *Smn* gene) are early embryonic lethal displaying enhanced neuronal death, indicating that the SMN is vital for normal cellular/neuronal function [304,305,306]. A lot of research has been put into defining the mechanisms of selective neuronal dysfunction in SMA. Lotti et al. identified an SMN-regulated U12 intron-containing gene, named Stasimon, responsible for motor circuit function. Restoration of Stasimon expression corrected deficits in neurotransmitter release at the neuromuscular junction (NMJ) and thereafter muscle growth in *Drosophila* SMN mutants as well as motor axon branching in SMN-deficient *Zebrafish*, suggesting that defective splicing of critical neuronal genes induced by SMN deficiency is responsible for motor circuit dysfunction [307]. Another finding is that neuritic properties are severely affected in SMA. *Smn*-deficient motor neurons display shorter neurites, fewer branches and poor terminal arborization with intermediate filament aggregates as well as impaired neurotransmitter release [308,309]. These effects have been, in part, attributed to an increase in the neuronal-specific profilin IIa (*PFN2*) isoform expression that drive Rho associated coiled-coil containing protein kinase (ROCK)/profilin IIa complex formation and subsequent disproportional induction of the RhoA/ROCK pathway resulting in altered cytoskeletal integrity [308,310,311,312]. Importantly, however, SMN has been proven, by multiple studies, to play a central role in regulating the localization of RNPs in axons and dendrites. SMN has a nuclear export signal and displays axonal localization utilizing microtubules for rapid bidirectional transport [313]. Knockdown of *Smn* in motoneurons resulted in transcripts related to immune functions and RNA splicing to be upregulated in the somatodendritic compartment while on the axonal side, transcripts associated with axon growth (*Apc*, *Dcx*) and synaptic activity (*Cpsf3*, *Syn3*, *Ank3*) were downregulated [314]. In addition, a global decrease in poly(A) mRNAs was observed in SMA axons that also resulted in deficiency of axonal protein synthesis [315]. SMN does not contain a characteristic RNA binding sequence. It has been proposed, however, that it contributes to the RNA transport in an indirect manner, through tight interactions with RBPs, modulating in this way both mRNA binding and transport [316,317]. HNRNPR, for instance, directly interacts with the 3′ UTR of *Actb* mRNA in axons and its depletion results in reduced presence of ACTB in the growth cone and consequently reduced axonal elongation [318]. Accordingly, SMN was shown to bind HNRNPR in axons and its depletion resulted in reduced *Actb* mRNA and protein staining in distal axons and growth cones that correlated with reduced axonal growth, suggesting that a complex of SMN with HNRNPR is required for *Actb* mRNA translocation [311]. Similarly, SMN was shown to bind ELAVL4 and ZBP1 in axons, and SMN knockdown in primary motor neurons resulted in a specific reduction of both HuD and ZBP1 proteins as well as poly(A) mRNA levels in the axonal compartment [92,288,315]. More recently, Donlin-Asp et al., using multiple biochemical and high-resolution microscopy assays, revealed that SMN deficiency leads to reduced binding of RBPs to their transcripts, smaller RNP granules are assembled, and there is reduced association of RNPs with microtubules and actin filaments [317].

Finally, SMN was shown to co-localize with TIA1/TIAR and G3BP in SGs during stress. Interestingly, suppression of SMN drastically reduced the cellular ability to form SGs in response to stress treatment and sensitized cells to noxious stimuli leading to cell death at much lower concentrations than in control cells [319], suggesting that it may represent a novel SG nucleator.

In conclusion, SMN functions as a molecular chaperone that facilitates spliceosomal snRNP assembly in the nucleus and through its interactions with multiple RBPs, in the cytosol, the assembly and transport of RNP granules.

## 5. Late-Onset Neurodegeneration

### 5.1. TAR DNA-Binding Protein 43 (TDP43)

TDP43 (transactive response DNA-binding protein 43 kDa or TARDBP), initially cloned and characterized as a transcriptional repressor, is a member of the hnRNP family, known for their role in RNA processing, that are localized in nucleus and are ubiquitously expressed [320]. TDP43 localizes to sites of transcription and splicing and is postulated to be part of the spliceosome [321], while it is absent from areas of silent heterochromatin [322]. TDP43 contains two RNA recognition motifs (RRM) and shows clear preference in binding to at least five UG repeats. TDP43 binds to more than 6000 RNA targets in the brain, roughly 30% of the total transcriptome, indicating that it has the potential to impact RNA metabolism on a global scale [323,324,325,326,327]. Most TDP43 binding occurs in introns (~70%) and to a lesser extent in 3′ UTR and non-coding RNA (~10%) [326]. Binding of TDP43 to deep intronic sequences (>2 kb from the nearest intro-exon junction) correlates positively with protein expression, suggesting that it may suppress cryptic splice site usage and/or promote mRNA stability [325,328,329]. TDP43 primarily influences alternative splicing in a position-dependent manner, similar to the other RBPs such as NOVA and ELAVL, leading to either exon inclusion or exclusion [326,330,331,332,333]. With respect to cytoplasmic TDP43, most binds on 3′ UTR sequences, indicating that it also regulates post-splicing events such as stabilization, transport or translation [326].

TDP43 is a central player in the pathogenesis of amyotrophic later sclerosis (ALS) and frontotemporal dementia (FTD, commonly referred as FTLD-TDP). In both neurons and glia of these patients, TDP43 is mislocalized from the nucleus to the cytoplasm, where it accumulates in abnormal phosphorylated, ubiquitinated and proteolytically-cleaved aggregates in 97% of ALS and 45% of FTD patients (sporadic and familial cases combined) [334]. Because this subcellular redistribution leads to nuclear depletion of TDP43, the pathogenic mechanism may involve loss of nuclear function, gain of cytoplasmic function or both. Currently, more than 50 missense mutations have been identified in the TDP43 gene accounting for an estimated 5% of familial ALS cases and <1% of sporadic cases [335,336,337]. Interestingly, nearly all of the disease-causing mutations are clustered in the Gly-rich domain that mediates protein–protein interactions with primarily other RBPs and hence affect TDP43 solubility, RNP transport, subcellular localization and recruitment to SGs [338,339,340]. Pathological TDP43 aggregates are also frequently present in other neurodegenerative diseases including AD, dementia with Lewy bodies (DLB) and Huntington’s disease (HD) (reviewed in [341]).

Overexpression and knockout studies have been employed both in vivo and in vitro to delineate the role of TDP43 in neurotoxicity. Mice overexpressing human wild-type or mutant (G348C and A315T) TDP43 displayed characteristics mimicking FTLD with ubiquitin positive inclusions (FTLD-U) and neurodegeneration. In particular, the transgenic mice exhibited impaired learning and memory, progressive motor dysfunction, and hippocampal atrophy accompanied by increased levels of gliosis [342,343]. Transgenic mice expressing TDP43^A315T^ showed age-dependent reduction in the development of basal mushroom spines that lead to lower efficacy of synaptic transmission within the motor cortex as determined by electrophysiology [344]. Interestingly, opposite results were observed after overexpressing the TDP43^Q331K^ mutation, which lead to increased excitatory synaptic inputs and dendritic spine densities [345]. Similar to overexpression studies, Cre/loxP mediated knockdown of TDP43 in motor neurons of mice revealed motor impairments, degeneration of large motor axons, grouped atrophy of the skeletal muscles, and denervation in the neuromuscular junction [346]. In *Drosophila*, overexpression of drosophila or human wild-type but not mutant TDP43 in vivo significantly increased dendritic branching of sensory neurons while loss of dTDP43 function, either in a genetic null background or through RNAi, decreased dendritic branching [347]. Overexpression of wild-type or mutant TDP43 in cultured motor neurons caused a severe impairment in axonal outgrowth, whereas TDP43 downregulation enhanced axonal branching [348]. Overall, these studies indicated that changes in TDP43 levels, whether it is an upregulation or downregulation, are accompanied by neuropathological alterations demonstrating a bidirectional role.

At the molecular level, TDP43 regulates an important array of genes that encode proteins involved in synaptic function [324,325,349,350]. In flies, loss of TDP43 reduced levels and altered the splicing of the calcium channel cacophony resulting in locomotion defects that were rescued by cacophony overexpression [351]. In the same animal model, the mRNA and protein levels ofSyntaxin 1A (STX1A, syx in *Drosophila*), a protein involved in synaptic vesicle fusion with the presynaptic membrane were reduced, suggesting that the function of TDP43 is to stabilize *Stx1a* mRNA and/or promote its translation [323]. Consequently, transgenic expression of STX1A in the TDP43 minus backgrounds was able to efficiently recover the locomotive defects [352]. Similarly, TDP43 was shown to bind *Map1b* mRNA and regulate its protein translation at NMJ. MAP1B is essential for microtubule assembly and therefore global synaptic mRNA transport in neurons. Loss of TDP43 in flies resulted in MAP1B downregulation at NMJ [353] that was accompanied by synaptic defects while elsewhere it was reported that wild-type or mutant TDP43 overexpression resulted in reduced MAP1B expression at the NMJ and increased expression in the cell body [354]. Additionally, TDP43 has been shown to bind to mRNA and regulate the expression of several proteins implicated in neurodegenerative diseases including ALS and FTD (e.g., FUS, TAU, Ataxin 2, and progranulin) [324,325,326,355,356,357,358]. More recently, disease-associated mutations were shown to increase TDP43 mitochondrial localization in neurons that deregulated mitochondria-transcribed mRNAs leading to mitochondrial dysfunction and neuronal loss [359]. Furthermore, using proximity-dependent biotin identification (BioID), it was revealed that aggregated and disease-linked mutant TDP43 triggered the sequestration and/or mislocalization of nucleoporins and transport factors, and interfered with nuclear protein import and RNA export in neurons. Importantly, nuclear pore pathology was found in brain tissues obtained from sporadic and familial ALS patients [360].

TDP43, like most RBPs, in response to stress is sequestered to SGs [339,361,362,363] where it co-localizes with its mRNA targets [364,365]. Furthermore, it has been shown to differentially regulate the expression of core RBPs of SGs, such as TIA1 and G3BP [366]. In TDP43 siRNA cells, TIA1 was upregulated while G3BP was downregulated. In addition, SGs were fewer and appeared smaller with a less-defined and more irregular morphology [366]. Moreover, following stress, disease-associated TDP43 mutants incorporated earlier into SGs, increased SG size and reduced their mobility [339,367].

Although its localization is mainly nuclear, TDP43 staining has been detected within synaptic terminals [350], along motor neuron axons [364] and in dendrites of neurons [368] indicating that it is involved in mRNA transport and translation. Indeed, TDP43 positive granules were shown to traffic bidirectionally in a microtubule-dependent manner along axons [364] especially upon stimulation of motor neurons with neurotrophin BDNF [348]. Interestingly, A315T and M337V mutations in TDP43 caused a decrease in the anterograde movement of TDP43 granules in axons and an increase in their retrograde movement, leading to an accumulation of mutant TDP43 granules in the proximal axon compartment and their depletion from axon terminals [364]. Further, Gopal et al., using super-resolution microscopy, revealed that TDP43 RNP granules in the proximal axon of cortical neurons were less circular and showed spiculated edges, whereas more distal granules were both more spherical and more dynamic suggesting that they are loosely attached to mRNAs as they reach the target site. On the other hand, RNP granules formed by ALS-linked mutant TDP43 were more viscous, bigger and exhibited disrupted transport dynamics [369].

Collectively, TDP43 has pleiotropic effects ranging from alternative splicing in the nucleus to synaptic mRNA delivery and local translation. Disrupting its expression or mutations will lead to neuronal loss and the development of neurodegenerative diseases such as ALS and FTD.

### 5.2. Fused in Sarcoma/Translocated in Liposarcoma (FUS/TLS)

Fused in Sarcoma/Translocated in Liposarcoma (FUS/TLS) can bind to single- and double- stranded DNA as well as RNA enriched for the GUGGU motif, however with limited specificity [370,371,372]. FUS can also directly associate with RNA polymerase II (RNAP2) and III (RNAP3) to regulate transcription [373,374,375] and polyadenylation site selection [373]. Furthermore, the association of FUS-RNAP2 with U1-snRNP ensures transcription-alternative splicing coupling [370,376]. FUS preferentially binds to 5′ and 3′ UTR of mRNAs and engages in nucleo-cytoplasmic shuttling [377,378]. Furthermore, FUS stimulates microRNA biogenesis by recruiting DROSHA co-transcriptionally [379]. Moreover, FUS is recruited to sites of DNA damage and plays an essential role in cellular recovery, through its interactions with CBP/p300 and HDAC1 [380,381].

Mutations in FUS have been linked to familial amyotrophic lateral sclerosis (ALS), with brains of affected patients demonstrating FUS-positive inclusions in the cytoplasm of degenerating neurons and glia as well as decreased levels of nuclear FUS [382,383,384]. Most FUS mutations cluster in the C-terminal nuclear localization signal and N-terminal prion-like domain deflecting FUS localization towards the cytoplasm and rendering it more prone to misfolding and aggregation [385]. Similar FUS pathology to ALS has also been observed in FTD, although no mutations in the FUS gene were required for the neurodegenerative phenotype [386,387].

The neurotoxic role of wild-type or mutant FUS has been widely demonstrated. Overexpression of FUS in mouse NSC34 motor neuron cells and primary cortical neurons induced increased cell death [388]. Moreover, mutant FUS transgenic rats developed progressive paralysis represented by severe axonopathy of motor neurons and denervation atrophy of skeletal muscles. In addition, they displayed a substantial loss of neurons in the cortex and hippocampus. This neuronal loss was accompanied by the appearance of ubiquitin bodies and glial reactivity [389]. Transgenic rats that overexpressed the wild-type human FUS were asymptomatic at younger ages, but showed deficits in learning and memory and a significant reduction in cortical and hippocampal neurons at later ages [389]. Similar results were obtained from the study of wild-type and mutant transgenic mice where motor neuron loss was associated with synapse disassembly at the neuromuscular junction [390]. Intriguingly, loss of FUS function is also detrimental. In *Drosophila* and Zebrafish, FUS knockdown is directly associated with neuronal cell death [391,392], while loss of FUS in mice causes perinatal lethality. Interestingly, conditional knockouts that eliminated FUS postnatally showed normal survival and had no effect on motor neuron survival or function [390]. In summary, these findings demonstrate that FUS-dependent motor degeneration is not due to loss of FUS function, but due to the gain of toxic properties conferred by overexpression and ALS-linked FUS mutations.

At the molecular level, analysis of ALS-linked FUS mutants has given useful information on the pathogenic mechanisms associated with FUS. Autophagy is the major degradation pathway for aggregate-prone proteins within lysosomes. Expression of FUS mutants was shown to impair autophagy in neuronal cells as exemplified by the accumulation of ubiquitinated proteins and autophagy substrates p62 and NBR1 [393]. An interesting insight arose from the observation that FUS interacts with SMN. Sun et al. revealed that FUS mutants display enhanced binding to SMN and deregulate its function, including loss of Gems and altered levels of small nuclear RNAs [394]. Moreover, the pathological mutations simultaneously decreased FUS binding to the U1 snRNP, resulting in global splicing disruptions that phenocopy a partial loss of FUS activity [394]. In addition, FUS mutants were shown to redistribute SMN towards cytosolic FUS aggregates, eventually decreasing available levels of axonal SMN. Overexpression of SMN rescued the axonal defects induced by mutant FUS, suggesting that FUS mutations cause axonal defects, in part, through SMN sequestration [395]. Furthermore, FUS mutants, but not wild-type FUS, assembled into perinuclear stress granules in response to oxidative stress or heat-shock indicating that they may influence SG dynamics [396].

FUS is involved in the transport of RNAs in dendrites via kinesin and dynein motors and can move into spines through the actin-based motor protein, myosin-Va [397]. Interestingly, FUS appears to be able to directly associate with the postsynaptic density (PSD) in an activity dependent manner [398,399]. In response to glutamate activation, FUS accumulates in the dendrites along with increased RNA content that include among others *Nd1-L* (*Ivns1abp*), an actin-stabilization protein, *ActB* and *Snap25* mRNAs [400,401,402]. In *Fus*-KO hippocampal neurons, following mGluR5-activation, *Nd1-L* transcripts localization was reduced in dendrites. These alterations were associated with more dendritic branches and immature spines [402]. Super-resolution microscopy has also revealed presynaptic localization of FUS in hippocampal neurons [403]. Intriguingly, FUS was found adjacent to the synaptic vesicle protein Synaptophysin 1 (SYP) and active zone protein Bassoon (BSN), but distant from the postsynaptic protein PSD95 [403]. The role of FUS at this location need to be further addressed. In NIH/3T3 cells, FUS was shown to be required for translation of localized mRNAs in APC-RNP granules found in pseudopodial protrusions [404]. Interestingly, overexpression of wild-type or FUS mutants resulted in formation of SG-like FUS granules at these sites, which recruited APC-RNPs and unexpectedly promotedtranslation of associated mRNAs [404]. Given that FUS associates with ribonucleoprotein complexes and polysomes in neurons, it is likely that FUS will also regulate dendritic and axonal translation. Future studies are required to confirm this notion.

Collectively, FUS seems to be instrumental in maintaining nuclear functions and axodendritic dynamics, the later based on RNA shuttling and transport properties.

### 5.3. TATA-Box Binding Protein Associated Factor (TAF) 15

TATA-box binding protein associated factor (TAF) 15, a member of the FUS, EWS and TAF15 (FET) protein family, binds RNAs enriched for the GGUAAGU motif, in vivo. Despite earlier reports suggesting a major role in alternative splicing, CLIP-seq analysis revealed that most binding sites are located in the 3′ and 5′ UTRs reflecting functions related to RNA turnover, transport and translation [378]. Nevertheless, a set of neuronal mRNAs encoding proteins with essential roles in synaptic activities has been shown to be dependent on TAF15 for alternative splicing events. These include *Grin1*, *Grin2a*, *Nrxn1*&*3*, *Nlgn1*, and *Pcdh9* [405]. Furthermore, TAF15 exhibited saw-tooth binding pattern on long introns, a pattern reminiscent of co-transcriptional splicing and/or stability [406], and genes downregulated upon loss of TAF15 contained exceptionally longer introns [378].

TAF15 was initially associated with chondrosarcoma and leukemia cancers due to the translocation and fusion of its N-terminal transactivation domain to the DNA-binding domain of NR4A3 or CIZ/MNP4 [407,408]. Following the discovery of the relation of TDP43 and FUS with ALS, TAF15 became a prime ALS candidate gene. Ticozzi et al. and Couthouis et al. both simultaneously identified several mutations that were strictly associated with patients with familial ALS [409,410].

TAF15 is pathogenic when expressed at high levels. In *Drosophila*, overexpression of wild-type TAF15 was associated with extensive neurodegeneration, while when TAF15 overexpression was targeted to motor neurons, progressive loss of motility was observed. Shorter fly lifespan was also seen when ALS-linked TAF15 mutants were employed compared to wild-type TAF15 [410]. These effects were associated with cytoplasmic inclusions of TAF15 in spinal cord motor neurons [410]. Analysis of post-mortem ALS patient tissues revealed that TAF15 was located in puncta mislocalized to the cytoplasm [410]. Similarly, in frontotemporal lobar degeneration (FTLD) with FUS inclusions, TAF15 co-localized with FUS in those inclusions [411]. TAF15 is inherently prone to aggregation as a result of the arginine and glycine rich (RGG) prion-like domain, a structure that characterizes other nucleating proteins of SGs [410]. In naive conditions, TAF15 is predominantly localized in the nucleus; however, it has been shown to co-localize with a minor subset of RNA granules in the cytoplasm, too [412]. Upon cellular stress, TAF15 travels to the cytosol and locates with FUS in SG-like structures, although its recruitment is not essential for SG formation [413,414].

In summary, TAF15 confers neurodegeneration in the nervous system when its levels are upregulated or mislocalized [410]. Although the specifics of TAF15 subcellular localization in neurons is still poorly described, knowing its high homology to FUS, it is expected to play a similar important role in axodendritic RNA transport [378].

### 5.4. T-Cell Intracellular Antigen (TIA) 1/TIA1-Related/Like Protein (TIAR)

T-cell intracellular antigen 1 (TIA1) and TIA1-related/like protein (TIAR) were originally discovered as components of cytotoxic T lymphocyte granules. They share 80% identity at the amino acid level [415,416] and have a broad tissue distribution, but they are predominantly expressed in brain, testis and spleen [416]. In naive conditions, they reside in the nucleus where they regulate transcriptional and post-transcriptional processes. TIA1 has been shown to bind both RNA polymerase II and T-rich stretches of single-stranded DNA to couple transcription and splicing of pre-mRNAs [417,418,419]. With respect to pre-mRNA splicing TIA1/TIAR bind to U-rich pentamers on mRNA proximal to the 5′ splice-acceptor sites in targeted introns, to enableU1 snRNP recruitment and the subsequent inclusion of the adjacent exon. It is estimated that approximately 15% of alternative exons are regulated by TIA1/TIAR binding to these sites [420,421,422,423,424]. In response to cellular stress, TIA1/TIAR translocate to the cytoplasm to suppress mRNA translation via binding to mRNA targets marked by U-rich motifs and nucleate SG formation [425,426,427]. Interestingly, while other RBPs (e.g., IGF2BP1 or HUR), which are dispensable for SG-assembly, are stably associated with SGs, TIA1/TIAR are only transiently associated with SGs, promoting SG-formation by constantly replenishing mRNPs [428]. Importantly, TIA1 oxidation by reactive oxygen species (ROS), such as H_2_O_2_, inhibits SG assembly and sensitizes cells to stress-induced apoptosis, suggesting that the suppression of SG formation by oxidative stress may underlie cell death pathways [429].

TIA1 is one of the most recent genes recognized for its role in ALS/FTD. Several disease-causing mutations have been identified that showed a consistent pathological signature with numerous round, hyaline, TDP43-positive inclusions in postmortem brains. These mutations changed the biophysical properties of TIA1 by delaying SG disassembly and promoting the accumulation of non-dynamic SGs that harbored TDP-43 [430].

The importance of TIA1/TIAR in animal physiology was revealed by in vivo studies of genetically modified mice deficient for TIA1 and or TIAR. Double KO mice were embryonic lethal. In the absence of TIA1 alone, half of the mice died perinatally. Those that remained alive developed inflammation in various tissues [431,432] in accordance with a well characterized role in modulating the expression of inflammatory proteins such as TNF-α, interleukin (IL) 1, IL6, matrix metalloproteinase (MMP) 13 and cyclooxygenase (COX) 2 [431,433,434,435,436]. In addition, transcriptome profiling of TIA1^−/−^ brain RNAome revealed alterations in the expression of cell cycle and apoptosis regulators as well as fat storage and membrane trafficking factors [437]. TIAR transgenic mice, similarly, displayed low transgene transmission associated with embryonic lethality starting at early post-implantation stages [438] pointing to the importance TIA1/TIAR expression for normal embryonic development.

Cell culture studies have provided additional evidence of the role of TIA1/TIAR in cell homeostasis. In HEK293 cells, double KO of TIA1 and TIAR increased target mRNA abundance proportional to the number of binding sites and also caused accumulation of aberrantly-spliced mRNAs, most of which were subject to nonsense-mediated decay. This compromised cell cycle progression and promoted apoptotic cell death [424]. Similarly, inactivation of TIA1 and TIAR in murine embryonic fibroblasts was associated with decreased proliferation, longer cell-cycle and increased cell size. Furthermore, TIA1/TIAR deficiency also led to metabolic deregulation, increased ROS levels and DNA damage, promoting a moderate increase in cell death [439]. TIA1 overexpression also affected mitochondrial biology. Specifically, it resulted in enhanced mitochondrial fission, ROS production and mitochondrial DNA damage [440,441]. TIAR protein expression was induced in wild-type mice after global cerebral ischemia and in cultured cortical neurons and astroglia after exposure to hypoxia. Immunohistochemical analysis revealed that TIAR protein expression was co-localized with DNA damage in neuronal cells in vivo, suggesting that it may be involved in neuronal cell death following ischemia [442].

One important aspect of TIA1 biology is its interaction with Tau (MAPT) protein. Tau plays a homeostatic role by binding and stabilizing the microfilament network in axons, promoting outgrowth and rapid axonal transport. In pathological conditions such as Alzheimer’s disease, Tau forms toxic oligomers and fibrils in foci located in the somatodendritic compartments and isolated processes of affected neurons [443]. Recently, Vanderweyde et al. reported that, in hippocampal neurons, Tau increased TIA1 localization in the soma and dendrites, and Tau accelerated SG formation and decreased TIA1 RNA granule movement. Conversely, TIA1 knockdown or KO inhibited Tau misfolding and associated toxicity in cultured hippocampal neurons, while TIA1 overexpression induced Tau misfolding and led to loss of axonal terminals and increased expression of apoptotic markers and toxicity [444]. The importance of the TIA1–Tau interaction for tauopathies was further explored using the PS19 transgenic Tau mice [445]. Reducing TIA1 decreased the number and size of granules co-localizing with other SG markers. Furthermore, decreasing TIA1 also inhibited the accumulation of Tau oligomers and increased neuronal survival as well as rescued behavioral deficits and lifespan. Collectively, these studies demonstrated the strong association of TIA1 with Tau pathophysiology [445].

In summary, TIA1/TIAR proteins have pleiotropic functions on embryonic development with an emphasis on cell cycle progression, inflammation and apoptosis. Maintaining physiological levels, like for other RBPs, is critical for proper cell function. Both TIA1/TIAR are core SG nucleating proteins. TIA1 strongly interacts with Tau to promote its aggregation in pathological conditions and Tau interferes with SG dynamics and TIA1 RNA granule transport.

## 6. Conclusions

The cellular and animal model paradigms discussed in this review reflect the increasingly compelling view that RBPs are critical mediators of neuronal function and dysfunction. This is reinforced, at the molecular level, by high-throughput sequencing studies, revealing that each RBP is implicated in the full spectrum of RNA processing events for hundreds of RNA targets in the cells in which they are expressed. These include alternative splicing, RNA editing, miRNA biogenesis, nucleo-cytoplasmic trafficking, axodendritic transport and local translation, all of which are indispensable for neuronal homeostasis and synaptic function.

**Figure 2:**
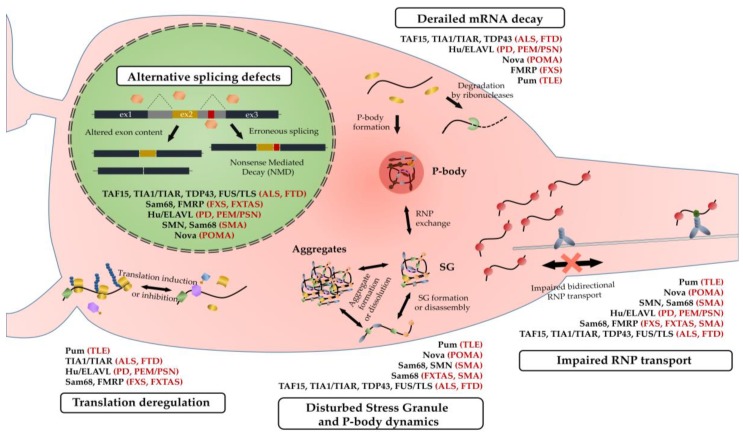
RNA metabolism in neurological disease. ALS: Amyotrophic Lateral Sclerosis, FTD: Frontotemporal Dementia, FXTAS: Fragile-X-Associated Tremor/Ataxia Syndrome, FXS: Fragile-X Syndrome, PD: Parkinson’s Disease, PEM/PSN: Paraneoplastic Encephalomyelopathy /Paraneoplastic Sensory Neuropathy, POMA: Paraneoplastic Opsoclonus Myoclonus Ataxia, SMA: Spinal Muscular Atrophy, TLE: Temporal Lobe Epilepsy.

A recurrent theme from all these studies is that deregulation of RBP levels, due to persistent cellular stress or mutations that alter subcellular distribution or physiological function, disrupts neuronal ribostasis and thereafter proteostasis, leading to neuronal dysfunction and the development of neurological symptoms such as intellectual disabilities, motor impairments and neurodegeneration (Figure 2). Importantly, in virtually all cases, synaptic function is compromised, representing an early step, and possibly the most critical, in neural network deregulation and subsequent disease development. This is, perhaps, not unexpected given that almost 50% of transcriptome is localized at the synapses [13] and that the axodendritic RNA transport and translation is mediated almost exclusively by the RBPs. Furthermore, RBPs bound to mRNA targets interact with numerous other RBPs and ribonucleoprotein species during RNP transport, a yet poorly characterized activity, that, if aberrantly perturbed, has the potential to quickly disperse and derail other subcellular processes. Another related finding is that highly polarized neurons with long axons, such as motor neurons, are particularly vulnerable to RBP deregulation.

The current challenge for disease therapy is to dissect those RBP–mRNA interactions, out of thousands in nucleus and cytosol, which are critical for neuronal homeostasis and find the means to preserve them. In addition, with the identification of hundreds of new RBPs, many of which lack conventional RBDs (reviewed in [446]), upcoming research should aim at analyzing their RNA targets, protein partners and specific housekeeping roles, hoping to complement existing knowledge on the regulation of gene expression in neurons.

## Figures and Tables

**Table 1:** Basic molecular functions and roles in neuronal homeostasis and disease.

Protein	Binding Motif(s)	Main Functions	Human-Associated Pathology	Rodent Knockouts
Pumilio	UGUANAUA	Translation repressor Induce SG assembly RNP granule transport	Temporal Lobe Epilepsy (TLE)	Reduced number of neural stem cells Enhanced dendritic outgrowth and arborization SCA1-like neurodegeneration Progressive motor dysfunction Impaired learning and memory
Staufen	RNA stem–loops	RNP granule transport Polarized mRNA transport Inhibit SG assembly Translation enhancement Staufen mediated decay (SMD)	-	Reduced number of neural stem cells Reduced dendritic tree arborization Fewer synapses Deficits in locomotor activity
Insulin-like Growth Factor 2 mRNA-Binding Protein (IGF2BP)	CAUH (H = A, U, or C) or CA-rich motifs	RNP transport Translation inhibition	-	Perinatal death Smaller animal size Compromised central and peripheral synaptogenesis PSC and NSC depletion and accelerated differentiation Axonal growth deficits after peripheral nerve injury
FragileX Mental Retardation Protein (FMRP)	TGGA	Transcriptional activation Alternative splicing Inhibit RNA A-I editing Translation inhibition RNP transport miRISC recruitment on target DNA damage response	Fragile-X Syndrome (FXS) Fragile-X-Associated Tremor/Ataxia Syndrome (FXTAS)	More and shorter dendritic spines and delayed maturation Reduced mobility of growth cones Deficits in learning and memory Hyperactivity
Src-Associated substrate in Mitosis of 68 kDa (Sam68)	UAAA or UUAA	Intracellular signaling adaptor Promotes translation Alternative splicing RNP granule transport Proapoptotic roles after injury	Fragile-X-Associated Tremor /Ataxia Syndrome (FXTAS) Spinal Muscular Atrophy (SMA)	Reduced NPC proliferation Fewer dendritic spines Motor coordination deficits
Cytoplasmic Polyadenylation Element Binding protein (CPEB)	UUUAU or UUUUAAU	Cytoplasmic polyadenylation Alternative Polyadenylation RNP granule transport Local translation Promote SG assembly	-	Impaired mitochondrial function Reduced dendritic mRNA transport Reduced theta burst-induced LTP Inability to extinguish memories Motor coordination deficits Motor learning delay
Neuro-Oncological Ventral Antigen (NOVA)	YCAY (Y is C or U)	Alternative splicing Alternative polyadenylation Inhibit nonsense mediated decay RNP granule transport	Paraneoplastic Opsoclonus Myoclonus Ataxia (POMA)	Nova1 KO: Death within 3 weeks of birth Axonal outgrowth defects Profound motor failure Nova2 KO: Death within 2 weeks of birth Aberrant migration of cortical and Purkinje neurons Axonal outgrowth defects No LTP following external stimulation Double KO: death soon after birth due to lack of lung motor innervation
Embryonic Lethal/Abnormal Vision-Like (ELAVL)	U-rich (with G or A intermittently)	Transcription rate Alternative splicing Alternative polyadenylation Compete with miRNA binding to mRNAs mRNA stability Translation enhancement RNP transport	Paraneoplastic Encephalomyelopathy/Paraneoplastic Sensory Neuropathy (PEM/PSN) Schizophrenia Parkinson’s disease (PD)	Neuron targeted HuR KO: motor neuron disease (poor balance, decreased movement and strength) HuC KO: Purkinje cell impaired functionality and morphology; Cerebral Ataxia; Epileptic seizures; Impaired spatial learning HuD KO: Impaired spatial learning; Lower levels of anxiety and activity; Predisposition towards auditory-induced seizures; Fewer differentiated neurons; Reduced axodendritic complexity; Cortical and motor deficits HuC/D doubleKO: perinatal death
Survival Motor Neuron (SMN)	not an RBP	snRNP assembly Alternative splicing RNP granule transport Axonal protein synthesis Induce SG assembly	Spinal Muscular Atrophy (SMA)	Embryos die prenatally Reduced association of RNPs with microtubules and actin filaments Shorter neurites, fewer branches and poor terminal arborization Enhanced neuronal death Smaller RNP granules
TAR DNA-binding Protein 43 (TDP43)	UG repeats	Alternative splicing mRNA stability RNP granule transport Induce SG assembly miRNA biogenesis Translation regulation Nuclear pore transport	Familial Amyotrophic Lateral Sclerosis (fALS) Frontotemporal dementia (FTD) Alzheimer’s Disease (AD) Dementia with Lewy bodies (DLB) Huntington’s Disease (HD)	Degeneration of large motor axons Skeletal muscle grouped atrophy Denervation of the neuromuscular junction
Fused in Sarcoma (FUS)	GUGGU	Alternative splicing Alternative polyadenylation Nucleocytoplasmic shuttling miRNA biogenesis RNP granule transport DNA damage response	Familial Amyotrophic Lateral Sclerosis (fALS) Frontotemporal dementia (FTD)	Perinatal lethality Enhanced neuronal cell death
TATA-box binding protein Associated Factor 15 (TAF15)	GGUAAGU	Alternative splicing RNA stability RNP granule transport	Familial Amyotrophic Lateral Sclerosis (fALS)	-
T-cell-restricted Intracellular Antigen 1 (TIA1) & TIA1-Related (TIAR)	T-rich motifs (DNA) U-rich motifs (RNA)	Alternative splicing Translation inhibition SG nucleation/assembly Inhibit nonsense mediated decay DNA damage response	Familial Amyotrophic Lateral Sclerosis (fALS) Tauopathies	TIAR: embryonic lethality TIA1: high rate of perinatal death, widespread inflammation TIA1/TIAR doubleKO: embryonic lethality

## Abbreviations

AD	Alzheimer’s Disease
ALS	Familial Amyotrophic Lateral Sclerosis
APA	Alternative Polyadenylation
ASD	Autism Spectrum Disorder
BDNF	Brain-Derived Neurotrophic Factor
BioID	Biotin Identification
CAMK2A	Calcium/Calmodulin-Dependent Protein Kinase Type II alpha
CPE	Cytoplasmic Polyadenylation Element
CPEB	Cytoplasmic Polyadenylation Element Binding
DLB	Dementia with Lewy Bodies
dsRBD	Double-stranded RNA-Binding Domain
eIF	Eukaryotic Initiation Factor
ELAV	Embryonic Lethal Abnormal Vision
FMRP	Fragile-X Mental Retardation Protein
FTD	Frontotemporal Dementia
FTLD	Frontotemporallobar Degeneration
FUS	Fused in Sarcoma
FXS	Fragile-X Syndrome
FXTAS	Fragile-X-Associated Tremor/Ataxia Syndrome
GMC	Ganglion Mother Cell
GWAS	Genome Wide Association Study
HD	Huntington’s Disease
HITS-CLIP	Cross-Linking Immunoprecipitation High-Throughput Sequencing
HNRNP	Heterogeneous Nuclear Ribonucleoprotein
IGF2BP	Insulin-Like Growth Factor-2 mRNA-Binding Protein
KH	(HNRNP) K-Homology Domain
KHDRBS1	KH Domain-Containing, RNA Binding, Signal Transduction Associated 1
KO	Knockout
LTD	Long-Term Depression
LTP	Long-Term Potentiation
miRNA	MicroRNA
MN	Motor Neuron
NMJ	Neuromuscular Junction
NOVA	Neuro-Oncological Ventral Antigen
NPCs	Neural Progenitor Cells
P-body	Processing Body
PD	Parkinson’s Disease
PEM/PSN	Paraneoplastic Encephalomyelopathy/Paraneoplastic Sensory Neuropathy
PND	Paraneoplastic Neurodegeneration
POMA	Paraneoplastic Opsoclonus Myoclonus Ataxia
PSD	Post-Synaptic Density
PUM	Pumilio
RBD	RNA-Binding Domain
RBP	RNA-Binding Protein
RNAi	RNA interference
RNP	Ribonucleoprotein
ROS	Reactive Oxygen Species
RRM	RNA Recognition Motif
Sam68	Src-Associated Substrate in Mitosis of 68 kDa
SCA1	Spinocerebellar Ataxia type 1
SCI	Spinal Cord Injury
SG	Stress Granule
SMA	Spinal Muscular Atrophy
SMD	Staufen-Mediated Decay
SMN	Survival Motor Neuron
snRNPs	Small Nuclear Ribonucleoproteins
STAR	Signal Transduction Activator of RNA
Stau	Staufen
TAF	TBP-Associated Factor
TDP43	Transactive Response DNA Binding Protein 43 kDa
TIA1	T-cell Intracellular Antigen 1
TIAR	TIA1-Related/Like Protein
TLE	Temporal Lobe Epilepsy
TLS	Translocated in Liposarcoma
UTR	Untranslated Region
